# Skin cancer diagnosis in renal transplant recipients during the Covid‐19 pandemic

**DOI:** 10.1002/ski2.69

**Published:** 2022-02-15

**Authors:** E. Keeling, J. Hynes, E. K. Pender, L. R. Griffin, M. E. Laing

**Affiliations:** ^1^ Department of Dermatology Galway University Hospital Galway Ireland; ^2^ Department of Dermatology Galway University Hospital, National University of Ireland Galway Ireland

Dear Editor,

1

The current Covid‐19 pandemic has caused significant disruption to dermatology services worldwide. Many dermatologists have been forced to move towards a new working environment, including a shift towards ‘teledermatology’/‘virtual reviews’ with the use of telephone calls, video calls and photographs. A number of reviews have demonstrated a reduction in the number of skin cancer referrals^1^ and diagnoses^1^, ^2^ during the pandemic. Guidelines have been published providing direction to dermatologists regarding the triage and management of skin cancer during this time.^3^, ^4^ Renal transplant recipients (RTRs) are known to have a significantly increased risk of developing skin malignancy secondary to chronic immunosuppression.^5^ There is a paucity of data pertaining to the impact of Covid‐19 restrictions on the diagnosis and management of skin cancers in this cohort.

We undertook a review of skin cancer diagnosis in RTR in a designated cancer centre during the current pandemic. In total, 167 RTRs attending the hospital were included in this review. A male predominance was noted, 113 males versus 54 females. The average age was 51 years, with a range of 17–82 years. Of this cohort, 33 patients had a history of previous skin cancer, with the majority representing previous squamous cell carcinomas. Of the 167 RTRs, 54 patients (32%) had been reviewed in the dermatology or plastic surgery department since January 2018. Since the beginning of the Covid‐19 pandemic (30 March 2020), 47 RTRs (28%) were reviewed in these departments. Of these, 36 (77%) were ‘in person’ reviews, facilitating full skin examination, while 11 (23%) were ‘virtual’ reviews.

A review of histologically proven skin cancers in RTR in the 12 months predating the start of the pandemic (31 March 2019 to 31 March 2020) compared with the first 12 months of the pandemic (01 April 2020 to 01 April 2021) was undertaken. Eighteen cases of non‐melanoma skin cancer (NMSC) were diagnosed in 12 RTRs in the year predating the pandemic, in comparison with 19 NMSCs diagnosed in 13 patients in the 12 months following the introduction of restrictions.

In contrast to previous reports, during the current pandemic, we note a slight increase in the number of skin cancers diagnosed in our RTR cohort. In view of the high‐risk nature of skin cancer in this cohort, the majority of RTRs attending our hospital were reviewed ‘in person’ as would have been standard practice prior to the pandemic, taking into account all safety precautions. We feel that face‐to‐face reviews were responsible for the stability in skin cancer diagnosis in our cohort. We suggest prioritization of ‘in person’ reviews for RTR attending for skin cancer assessment given their increased risk and poorer prognosis associated with skin malignancy.

## CONFLICTS OF INTEREST

The authors have no conflicts of interest to declare.

## AUTHOR CONTRIBUTIONS

**E. Keeling:** Conceptualization; Formal analysis; Investigation; Methodology; Project administration; Writing – original draft; Writing – review & editing. **J. Hynes:** Data curation; Formal analysis; Investigation; Methodology; Writing – review & editing. **E. K. Pender:** Formal analysis; Investigation; Methodology; Writing – review & editing. **L. R. Griffin:** Formal analysis; Investigation; Methodology; Writing – review & editing. **M. E. Laing:** Conceptualization; Investigation; Methodology; Supervision; Writing – review & editing.

## Data Availability

Research data are not shared.
